# Associations between Handedness and Cerebral Lateralisation for Language: A Comparison of Three Measures in Children

**DOI:** 10.1371/journal.pone.0064876

**Published:** 2013-05-30

**Authors:** Margriet A. Groen, Andrew J. O. Whitehouse, Nicholas A. Badcock, Dorothy V. M. Bishop

**Affiliations:** 1 Behavioural Science Institute, Radboud University Nijmegen, Nijmegen, The Netherlands; 2 Telethon Institute for Child Health Research, Centre for Child Health Research, University of Western Australia, Perth, Australia; 3 School of Psychology, University of Western Australia, Perth, Australia; 4 ARC Centre of Excellence in Cognition and its Disorders, Department of Cognitive Science, Macquarie University, Sydney, Australia; 5 Department of Experimental Psychology, University of Oxford, Oxford, United Kingdom; University of Texas at Dallas, United States of America

## Abstract

It has been known for many years that hand preference is associated with cerebral lateralisation for language, but the relationship is weak and indirect. It has been suggested that quantitative measures of differential hand skill or reaching preference may provide more valid measures than traditional inventories, but to date these have not been validated against direct measures of cerebral lateralisation. We investigated the associations of three different handedness assessments; 1) a hand preference inventory, 2) a measure of relative hand skill, and 3) performance on a reaching task; with cerebral lateralisation for language function as derived from functional transcranial Doppler ultrasound during a language production task, in a group of 57 typically developing children aged from 6 to 16 years. Significant correlations between cerebral lateralisation for language production and handedness were found for a short version of the inventory and for performance on the reaching task. However, confidence intervals for the correlations overlapped and no one measure emerged as clearly superior to the others. The best handedness measures accounted for only 8–16% of the variance in cerebral lateralisation. These findings indicate that researchers should not rely on handedness as an indicator of cerebral lateralisation for language. They also imply that lateralisation of language and motor functions in the human brain show considerable independence from one another.

## Introduction

Neuropsychologists have long been interested in handedness as a possible indirect measure of cerebral lateralisation for language function. Indeed over 90% of right-handers have language skills lateralised to the left hemisphere [1]–[6]. However, this is also the case for about 67–85% of left-handers [1]–[6]. Even after the development of accurate and reliable neuroimaging methods, hand preference is still often used as a proxy for cerebral lateralisation, presumably because it is a cheap and very accessible measure. A quick search using the Web of Knowledge [7] shows that 469 original articles on cerebral lateralisation, published between 2000 and 2012, cite one immensely popular handedness inventory, the Edinburgh Handedness Inventory (EHI) [8]. Of these papers, only 217 also included a neuroimaging technique (i.e., magnetic resonance imaging, computer tomography, positron emission tomography, magnetoencephalography, electroencephalography, functional transcranial Doppler ulstrasound or functional near-infrared spectrography; n = 178) or another behavioural measure of cerebral lateralisation (i.e., dichotic listening or visual half-field technique; n = 39). This suggests that 252 original articles on cerebral lateralisation (54%), published at a time when neuroimaging techniques were widely available, used hand preference as the main measure.

Assessing handedness is not as straightforward as it might seem. Some researchers simply categorized people as left-handed or right-handed based on the hand used to hold a pen when writing [9]. One objection to this definition of handedness is the strong influence of teaching on writing, with explicit discouragement of left-handedness in some cultures. Furthermore, writing hand cannot be assessed in young children or illiterate adults. In addition, a simple dichotomy may be too insensitive: Whereas some people exclusively use one hand, others use one hand for some activities and the other hand for other activities [10]. In an attempt to refine handedness assessments, inventories have been developed where preferences for a wide range of activities are combined [8], [11]–[13]. But several problems with hand preference inventories have been identified: 1) the selection of activities is arbitrary; 2) activities included are influenced to varying degrees by experience and social pressure; and 3) data from preference inventories usually result in highly skewed distributions, rendering parametric statistical techniques invalid. Finally, although one aim of the hand preference inventory was to move away from treating hand preference as categorical, in practice, inventory data more often than not are used to subdivide participants into different hand preference groups [10]. Apart from the issue that there is no agreement as to where cutoffs should be placed to create hand preference groups, it has been argued that hand preference is a quantitative trait, and that relative differences in motor skill between the left and right hand underlie preference [14], [15]. Following this line of reasoning, several proficiency measures have been used to measure handedness, such as peg moving [16], finger tapping [17] or dotting within a boundary [18]. Advantages of these performance measures are that they result in a quantitative measure of relative hand skill that is normally distributed. However, performance tasks suffer from problems 1) and 2) as much as inventories, and additionally, are more difficult to administer in that they require equipment and are often given on an individual basis. A different approach to measure handedness has been to assess preference rather than relative skill, but using a behavioural continuum rather than an inventory [19], [20]. The main idea in these studies is that a person who has a strong preference for one hand will keep using that hand to carry out a uni-manual activity, even if it is awkward to do so. Specifically, people are asked to reach across different locations in extrapersonal space in order to move pegs [20] or pick up cards [19]. Although the motor movement involved in performing an activity on the left of the midline might be easier when carried out with the left hand, right-handers usually reach across the midline and use the right-hand instead.

The aim of the current study was to determine the validity of different handedness assessments as an indirect measure of cerebral lateralisation for language. We investigated the associations of three different handedness assessments with an independent measure of cerebral lateralisation for language function in a group of 57 typically developing children. The Edinburgh Handedness Inventory [8], Annett’s peg-moving task [16], and the Quantification of Hand Preference (QHP) task [19] were used to assess handedness. Based on a factor analysis, Bryden [13] concluded that a shortened version of the EHI yields more useful results. Therefore we also included a handedness quotient, based on a short version of the EHI. It should be noted that these three different measures of handedness make different assumptions as to the relevant dimension that underlies the association between handedness and cerebral lateralisation for language. The inventory and the QHP task measure preference, whereas the peg-moving task measures relative skill. While the inventory summarizes the consistency of hand preference across different activities, the QHP task uses a behavioural continuum to characterize individual variation in hand preference. Functional transcranial Doppler ultrasound (fTCD) during an animation description task [21] was used to measure cerebral lateralisation for language. In the last decade, this noninvasive and relatively inexpensive technique has been shown to be a reliable method for determining cerebral lateralisation of function [22], well suited for use with children [21], [23], [24].

## Materials and Methods

### Participants

Participants were 57 typically developing children (32 girls, 25 boys) across three age bands 6–8 (*M* = 6.97 years, *SD* = 0.40 years), 9–11 (*M* = 10.79 years, *SD* = 0.43 years) and 13–16 years of age (*M* = 14.21 years, *SD* = 0.81 years), recruited from schools around Oxfordshire, UK. Five additional children were dropped from the study because of noisy fTCD recordings (two six-year-olds, one eight- and one ten-year-old), or because no hand preference data were collected (one 16-year-old). Results on cerebral lateralisation for language production (and visuospatial memory), and on cognitive and language tests from this sample have previously been reported, confirming no association between language lateralisation and age or gender in this sample [25]. Participants were without any history of neurological disorder and with normal or corrected-to-normal vision. Parents of the participants confirmed that no child had a diagnosis of a neurodevelopmental disorder, such as autism, specific language impairment or dyslexia, and that English was the main language spoken at home. The sample showed average performance on standardized measures of non-verbal cognitive ability (Leiter International Performance Scale-Revised, [26], *M = *102.07, *SD* = 14.16, Range 71–131) and vocabulary (British Picture Vocabulary Scale, [27], *M = *108.60, *SD* = 11.16, Range 82–132).

### Ethics Statement

Parental written consent was obtained for all participants. The project was approved by the Central University Research Ethics Committee of the University of Oxford and is in accordance with the WMA Declaration of Helsinki for experiments involving humans.

### Handedness Measures

Three different measures of handedness were obtained. *The Edinburgh Handedness Inventory* (EHI) [8] assesses the hand used during the following 10 activities: writing, drawing, throwing, using a toothbrush, using a knife (without a fork), using a spoon, holding a broom (upper hand), striking a match (hand holding the match), using scissors, and opening a box (hand used to hold the lid). The items “striking a match” and “using scissors” were considered inappropriate for young children. Instead, we asked children which hand they use to deal playing cards. We recorded whether they used the left or the right hand for each activity. A handedness quotient was calculated ((R−L)/(R+L)*100), with positive numbers indicating right-handedness, and negative numbers left-handedness. Following Bryden [13] we also calculated a shortened handedness quotient, based on the items writing, drawing, throwing, using a toothbrush and dealing playing cards (the latter as a substitute for the item ‘using scissors’).

Relative hand skill was assessed with Annett’s *Peg-moving task* [16]. This involved moving 10 pegs as quickly as possible from the back row to the front row of a pegboard, starting with the preferred hand and then alternating hands until three trials had been completed with each hand. The pegs were 5.1 cm long, and made of dowelling rod that was 1 cm in diameter. A measure of relative hand skill was calculated ((L−R)/(L+R)*100), with negative numbers indicating a faster performance (indicating higher skill) on left-hand trials and positive numbers a faster performance on right-hand trials.

Finally, the *Quantification of Hand Preference* task (QHP) [19] was given. This task provides a behavioral measure of hand preference. In this task, stacks of three cards with brightly coloured pictures were placed in seven spatial locations (approximately 30 degrees apart) along a semi-circle, on a table, within the child’s reach. The child was seated in the center of the semi-circle and asked to pick up a specific card and place it in a box located directly in front of them, without time constraints. The card order was random, but the sequence of positions was the same for all participants. The child was not informed of the experimental interest in hand preference, and treated the task as one of finding the named picture. The dependent variable was a laterality quotient (LQ), calculated by subtracting 0.50 from the proportion of right-hand reaches. This score ranged from +0.50 for participants reaching exclusively with the right hand through 0 for children who did not show a preference to −0.50 for those reaching exclusively with the left.

### Apparatus

Blood flow velocity through the right and left middle cerebral arteries was measured with a Doppler ultrasonography device (DWL Multidop T2: manufacturer, DWL Elektronische Systeme, Singen, Germany). Participants were fitted with a flexible head-set, which held in place a 2-MHz transducer probe over each temporal skull window. The experimental paradigm was controlled by Presentation Software (Neurobehavioral Systems) on a Dell laptop computer, which sent markers to the fTCD to denote the start of each epoch.

### Experimental Paradigm

An animation description paradigm, described in detail elsewhere [21], was used to elicit spoken language. In short, participants watched clips from a children’s cartoon which included sounds but no speech. Each trial started with the 12 s cartoon clip, which the participant was asked to watch silently. Then a response cue indicated the start of a 10 s animation description period during which the participant described what had been seen in the previous clip. This was followed by an 8 s silent rest period. A maximum of 30 clips was used. Note that during the pre-speaking baseline period participants watched the animation. We had previously established in pilot studies that there was no evidence of lateralised activation while participants passively watched these animations.

### Procedure

Participants were tested in a quiet laboratory, a separate room in their school, a testing van or at their home. All participants completed the handedness, cognitive and language tests in the first testing session and the language production paradigm in the second session.

### Functional Transcranial Doppler Analysis

Data from each fTCD paradigm were analysed using the dopOSCCI toolbox [28], which summarises fTCD data in MATLAB (Mathworks Inc., Sherborn, MA, USA). The following steps were carried out: 1) the blood ﬂow envelope from each probe was downsampled to 25 Hz, 2) heart beat activity was removed by determining local peaks in the signal from the left probe and using the heart cycle integration described by [29], 3) in order to control for global differences in recorded velocity, unrelated to the task, between the left and the right probe, blood ﬂow velocity was normalised to a mean of 100% on a trial-by-trial basis. Time-locked epochs were then averaged, after rejecting epochs with unusually high or low levels of activity (±40% of the average blood flow velocity). The mean difference curve for left and right channels was corrected to give a mean value of zero over a baseline period of 10 s prior to the presentation of the stimulus.

A laterality index (LI) was calculated as the mean blood flow velocity difference in a two second window centred on the peak difference value during the period of interest. The period of interest was based on previous work [30] and occurred during the speaking phase of the language production paradigm (4–14 s after onset of the cue to speak). A positive LI indicated greater left than right hemisphere activation, with a negative index signifying the reverse. As well as computing an LI, we categorised children as being left- or right-lateralised or showing bilateral activation, determining whether the 95% confidence interval of that individual’s LI overlapped with zero. Trials during which the participant was not “on task” (e.g., not paying attention, talking during the baseline) were excluded from the analysis. Only children who had at least 12 accepted epochs were included in the analysis (*M* = 18.25, *SD* = 2.82).

## Results

The majority (n = 44, 77%) of children showed left-lateralised activity for language production. Among the remaining children, 10 (18%) showed right-lateralised activity, whereas three children (5%) showed bilateral activity for language production. Descriptive statistics for all measures can be found in Table 1.

**Table 1:** Descriptive statistics.

	*M*	*SD*	*Mdn*	Range
Cerebral lateralisation for language production	2.00	3.20	2.95	−6.31–7.77
Edinburgh Handedness Inventory	63.57	44.53	77.78	−77.78–100.00
Edinburgh Handedness Inventory (short)	73.52	46.29	100.00	−100.00–100.00
Peg-moving task	4.65	5.67	4.66	−8.02–14.30
Quantification of Hand Preference task	.26	.23	.31	−.50–.50

In previous studies, using fTCD, handedness has often been examined categorically and a (trend for a) higher incidence of atypical lateralisation of language function has been reported in adults who are not right-handers as indicated by their performance on an inventory [3]–[5]. As a first step, we performed such an analysis on the current data. If we compare the number of right-handed children (as defined by a score of 40 or above on the full EHI [8]) with the number of children who are not right-handed, across language lateralisation groups (left vs. other), we obtain a similar result. As can be seen in Table 2, 50% of children who are not right-handed, but only 16% who are right-handed show atypical lateralisation of language function (bilateral or right-lateralised activity). This results in a significant association between handedness group and lateralisation group (Fisher’s Exact test, *p* = .020).

**Table 2:** Crosstabulation of participants’ cerebral lateralisation for language, based on LIs and 95% confidence intervals, as a function of handedness as derived from the full Edinburgh Handedness Inventory. Participant numbers are presented, with proportion of participants within each handedness category in parentheses.

Language	Handedness	
	Other	Right
Other	6 (50)	7 (16)
Left	6 (50)	38 (84)

**Figure 1:**
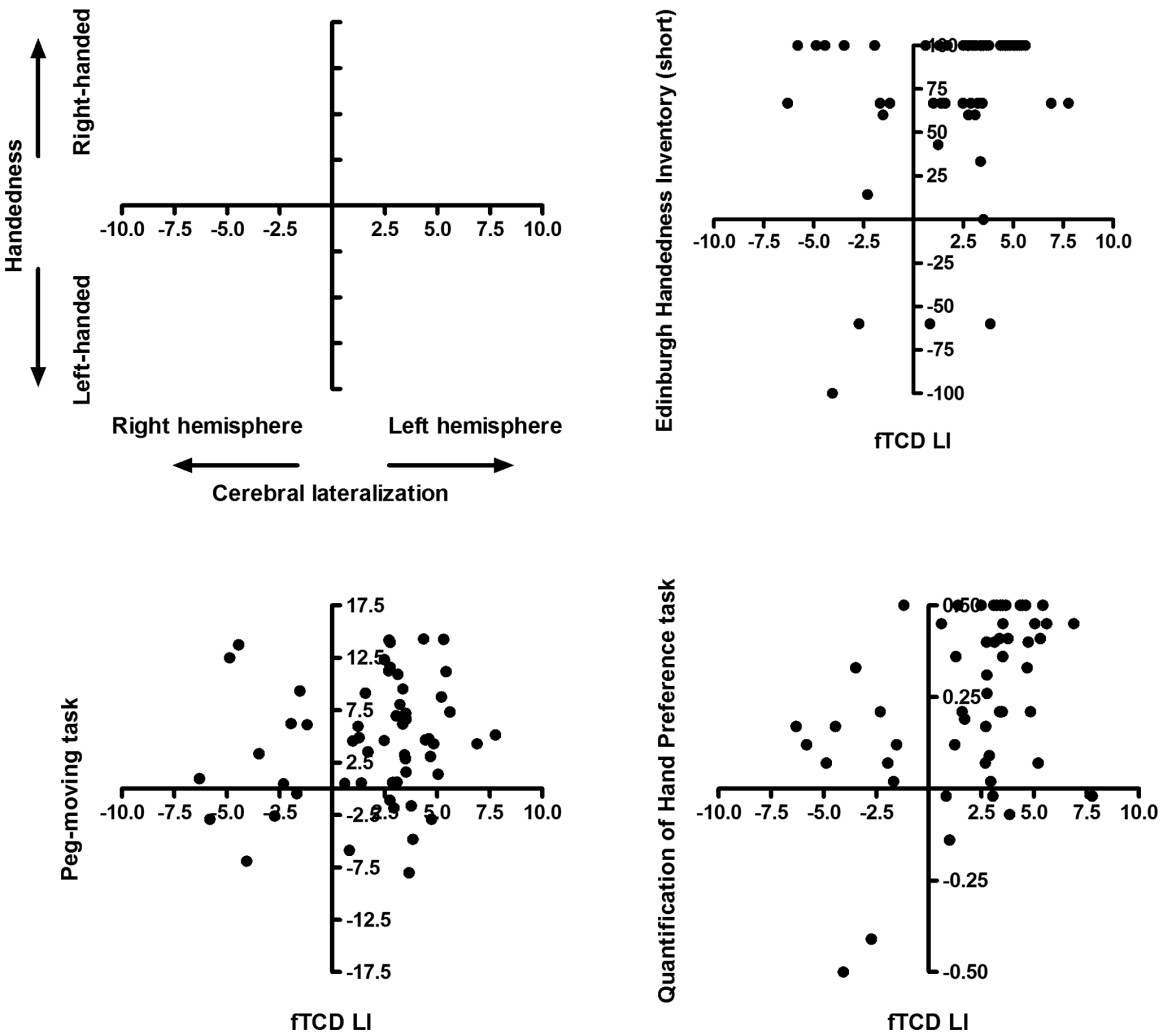
Associations between hand preference measures and cerebral lateralisation for language production. Scatterplots of performance on the short version of the Edinburgh Handedness Inventory (top right panel), Peg-moving task (lower left panel), and Quantification of Hand Preference task (QHP; lower right panel) on the y-axis and cerebral lateralisation for language production as indicated by the lateralisation index (LI) as derived from functional transcranial Doppler ultrasound (fTCD) on the x-axis.

There are, however, two problems with such a categorical analysis of the association between handedness and cerebral lateralisation for language. Firstly, there is no agreement as to where cutoffs should be placed to create handedness groups. Secondly, it has been argued that hand preference is a quantitative trait and treating it as a dichotomy results in the loss of important information on variation within groups of right- or left-handers [10], [11], [31]. Therefore, we calculated correlations between the handedness measures and cerebral lateralisation for language production. Because none of the measures, except relative hand skill derived from the Peg-moving task, were normally distributed, we report non-parametric correlation coefficients in the cells above the diagonal in Table 3. Weak to moderate correlations between the EHI (short) and the Peg-moving task (.28), and the EHI (short) and the QHP task (.24) were found. More interestingly, handedness as measured by the short version of the EHI (.29) and the QHP task (.40), but not the other measures, correlated significantly with cerebral lateralisation for language production. Scatterplots with handedness on the y-axis and cerebral lateralisation for language production on the x-axis are shown in Figure 1. Given the small number of left-handers included in the sample, these correlations might be unduly influenced by these few children. Additionally, if handedness is a quantitative trait, a measure must differentiate degrees of handedness within a group of right-handers or left-handers as well as show differences between left- and right-handers [19], [32]. We therefore calculated correlation coefficients when only including children with positive handedness (EHI short) or laterality (QHP task) quotients. In this case, hand preference as measured by the EHI (short) did not correlate significantly with cerebral lateralisation for language (ρ (53) = .24, *p* = .086, 95% CI: −.04–.48), but performance on the QHP task did (ρ (50) = .44, *p* = .002, 95% CI:.17–.60). A similar pattern of results is obtained if consistent right-handedness is defined as having positive scores on all three handedness measures (EHI short, Peg-moving task, and QHP task), as suggested by one of the reviewers. Hand preference as measured by the EHI (short) did not correlate significantly with cerebral lateralisation for language (ρ(42) = .30, *p* = .051, 95% CI: −.01 −.56), but performance on the QHP task did (ρ(42) = .38, *p* = .014, 95% CI:.07 −.62). Finally, it has been suggested that not the direction, but the strength or degree of handedness might be a more relevant marker of cerebral lateralisation [33]. This idea is supported by a functional magnetic resonance imaging study that reported an association between the amount of activation in motor cortex and degree of handedness, with people with a stronger hand preference showing less activity in ipsilateral motor cortex when using the dominant hand, regardless of direction of handedness [31]. Similarly, the size of the corpus callosum – often advocated as an anatomical marker of functional lateralisation [34], [35] – has been found to vary with degree, rather than direction of handedness [36]. To investigate whether consideration of degree of handedness might lead to a different pattern of associations, we computed correlations between cerebral lateralisation for language production and the absolute handedness quotient (EHI and EHI short), measure of relative hand skill (Peg-moving task) and laterality quotient (QHP task). Again, we found a significant correlation for the QHP task (ρ(57) = .28, *p* = .032, 95% CI:.02 −.51), but not for the measures based on the inventory (EHI: ρ(57) = .10, *p* = .465, 95% CI: −.17 −.36; EHI short: ρ(57) = .22, *p* = .096, 95% CI: −.05 −.46) or the Peg-moving task (ρ(57) = .11, *p* = .404, 95% CI: −.16 −.37).

**Table 3:** Non-parametric correlations, Spearman’s rho, between cerebral lateralisation for language production and different handedness measures for the full sample (n = 57) are presented in the cells above the diagonal; the 95% confidence intervals of the correlations are in the cells below the diagonal.

	1.	2.	3.	4.	5.
1. Cerebral lateralisation for language	−	.16	.29*	.13	.40**
2. Edinburgh Handedness Inventory	−.11–.41	−	.72**	.20	.18
3. Edinburgh Handedness Inventory (short)	.02–.52	.57–.83	−	.28*	.24∧
4. Peg-moving task	−.14–.38	−.07–.44	.01–.51	−	.19
5. Quantification of Hand Preference task	.15–.60	−.09–.43	−.03–.48	−.08–.43	−

∧*p*<.1;

* *p*<.05;

** *p*<.01.

These results seem to suggest that performance on the QHP, but not the other handedness measures, is associated with cerebral lateralisation for language production. But, as can be seen in the cells below the diagonal of Table 3 and in the text, the 95% confidence intervals of the correlations overlap. As such, we cannot conclude that handedness as measured by the QHP task is a better indicator of cerebral lateralisation than the other handedness measures, although it does appear to be more sensitive to variation in hand preference across the continuum.

## Discussion

It has long been known that a relationship between handedness and cerebral lateralisation for language exists, albeit a weak and indirect one. Nevertheless, handedness was used as a proxy for lateralisation in 54% of original research papers published between 2000 and 2012 on cerebral lateralisation. In the current paper we investigated associations of three different handedness measures with an independent measure of cerebral lateralisation for language derived from fTCD during a language production task in children.

As was found in previous fTCD research [3]–[5], when analyzing the results categorically (right-handed vs. not right-handed) we found an association between handedness group and lateralisation group. Children who are not right-handed (as indicated by their performance on the inventory) more often showed atypical lateralisation for language production. This result is also in agreement with results obtained from patient studies [1], [2] and a study in children using fMRI [6].

However, given that no consensus on the definition of handedness groups exists and handedness has been argued to be a quantitative trait [10], [11], [31], we calculated correlations. Significant correlations between cerebral lateralisation for language production and handedness were found for a short version of the inventory and for performance on the QHP task. Considering associations with degree of handedness, irrespective of direction, resulted in a significant correlation with performance on the QHP task, but not the other handedness measures. But, as confidence intervals for the correlations overlapped, we cannot conclude that one handedness measure emerged as clearly superior to the others.

It is important to note that only a small number of left-handers was included in the sample. Although this reflects the distribution of handedness in the population, this is a limitation of the current study. It is reassuring that the QHP task has previously been shown to be sensitive to differences in left-handedness [37], but oversampling left-handed children in a future study would be needed to confirm that these relationships hold for left-handed as well as right-handed children.

Although no one handedness measure appeared as clearly superior to the others, it is noteworthy that the QHP, which showed the strongest association with cerebral lateralisation in this sample, was a better predictor than a handedness inventory in other contexts. First, performance on the QHP task has been found to be more sensitive than data from inventories in distinguishing children with language difficulties from typically developing children and children with general delays [38], [39]. In these studies, children with specific language difficulties were less likely to use the preferred hand to cross the midline. Second, a modest but significant heritability has been found for a measure from the QHP task that reflected the tendency to persist in using the preferred hand (whether left or right) across the midline using twin data, but no such effect was found for performance on a handedness inventory [39]. Together this suggests that measuring preference (rather than relative hand skill) across a behavioural continuum (rather than across different activities) shows promise as a sensitive indicator of handedness.

Concluding, while the results for the QHP measure are more encouraging than for other measures, the data indicate that none of the handedness measures work well as a proxy for cerebral lateralisation for language. Even handedness measures that did show an association explain just 8% (EHI short) to 16% (QHP) of the variance in cerebral lateralisation for language production. It seems that if researchers are looking to include participants showing a specific (typical or atypical) pattern of language lateralisation, a behavioural screening measure based on the visual half field technique is a much better predictor than handedness [40]. Additionally, our findings join a growing body of work that suggests that lateralised functions in the human brain – in this case language and motor functions – are not determined by a single common cause, but show considerable independence from one another [5].
